# Growth, body condition and contest performance after early‐life food restriction in a long‐lived tropical fish

**DOI:** 10.1002/ece3.7867

**Published:** 2021-07-24

**Authors:** Angelika Ziegelbecker, Kristina M. Sefc

**Affiliations:** ^1^ Institute of Biology University of Graz Graz Austria

**Keywords:** body size, cichlid fish, compensatory and catch‐up growth, contest competition, growth trajectory, juvenile nutrition, life history, stress response

## Abstract

Adverse conditions during early life can cause lasting body size deficits with effects on social and sexual competition, while an accelerated growth response can allow animals to catch up in body size but can be physiologically costly as well. How animals balance growth deficits and growth compensation is predicted to depend on the effects of each on lifetime fitness. We investigated the effects of experimental early‐life food restriction on growth, body condition, and adult contest competition in a cichlid fish (*Tropheus* sp.). Their longevity and aseasonal breeding suggest that, with view on lifetime reproductive success, temporarily growth‐restricted *Tropheus* should rather invest extra time in reaching competitive body size than risk the potential costs of accelerated growth. However, size‐selective predation pressure by gape size‐limited piscivores may have favored the evolution of an accelerated growth response to early‐life delays. Experimentally food‐restricted fish temporarily reduced their growth rate compared to a control group, but maintained their body condition factor at the control level throughout the 80‐week study period. There was no evidence for an accelerated growth response following the treatment, as the food‐restricted fish never exceeded the size‐specific growth rates that were measured in the control group. Food‐restricted fish caught up with the body size of the control group several months after the end of the treatment period and were as likely as control fish to win size‐matched contests over territories. Regardless of feeding regime, there were sex‐specific differences in growth rates and in the trajectories of condition factors over time. Females grew more slowly than males but maintained their condition factors at a high level throughout the study period, whereas the males' condition factors declined over time. These differences may reflect sex‐specific contributions of condition and body size to adult fitness that are associated with female mouthbrooding and male competition for breeding territories.

## Graphical abstract

We investigated how long‐lived tropical cichlid fish (*Tropheus* sp.) balance costly growth deficits and costly growth compensation after early‐life food stress. Monitoring body sizes, growth rates, and condition factors of juvenile fish during and after an experimental food restriction, as well as performance in contest competition later in life, we showed that the fish caught up by prolonged growth at normal size‐specific rates without suffering a deterioration of any of the fitness‐related traits that were monitored in this experiment. We detected sex‐specific differences in growth rates and condition factors, which likely reflect the sex‐specific contributions of condition and body size to adult fitness.

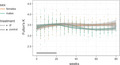

## 1 INTRODUCTION

In many animals, performance in contest competition regulates access to resources and thus can affect lifetime fitness (e.g., Kelly, 2008). Success in contest competition is often strongly dependent on body size, but also influenced by the expression of armaments and ornaments, current dominance status, cognitive abilities, and physiological condition (Allen & Krofel, 2017). Additionally, there is evidence that the competitive performance of adults reflects not only their current physical state, but also conditions experienced during early development (Metcalfe & Monaghan, 2001; Monaghan, 2008). Young animals can be exposed to a broad range of stressors, including food shortage, infections, as well as adverse social environments and unfavorable temperature, oxygen, and light conditions (Eyck et al., 2019; Metcalfe & Monaghan, 2001; Vindas et al., 2018). Across diverse vertebrate and invertebrate taxa, food‐deprived juveniles or larvae show significantly reduced growth rates compared to control groups (e.g., Ali et al., 2003; Dmitriew & Rowe, 2011; Richner et al., 1989) and are less immunocompetent (Clough et al., 2016), less competitive (Richner et al., 1989), and suffer higher predation risk (Hoey & McCormick, 2004; Sogard, 1997). The body size deficits developed during periods of retarded growth may be leveled out by accelerated (faster than normal for a given body size) or prolonged growth when conditions improve (Hector & Nakagawa, 2012; Metcalfe & Monaghan, 2001). However, other food stress‐induced deleterious effects may persist into adulthood or even be propagated by the costs of accelerated growth (Dmitriew, 2011; Hector & Nakagawa, 2012; Metcalfe & Monaghan, 2001). Reported side effects of fast growth range from elevated oxidative damage (Monaghan et al., 2009) and risk of developmental abnormalities (Ali et al., 2003; Hector & Nakagawa, 2012) to increased telomere shortening (McLennan et al., 2016) and reduced life span (Inness & Metcalfe, 2008; Metcalfe & Monaghan, 2003). Poor feeding conditions during juvenile development were shown to affect adult immune function (Butler & McGraw, 2012; Mugabo et al., 2010) and body mass loss in times of adult food restriction (Dmitriew & Rowe, 2011; Krause et al., 2009). Other studies described adult deficits in exploratory behavior (Krause & Naguib, 2011), learning abilities (Kotrschal & Taborsky, 2010), and locomotor performance (Álvarez & Metcalfe, 2007; Lee et al., 2016) in consequence of nutritional stress during early life. Such lingering effects can in one way or another have repercussions on contest performance and dominance status later in life (Richner et al., 1989; Royle et al., 2005).

Unlike determinate growers such as most insects and endotherm vertebrates, most fish continue their structural growth throughout much of their life, although growth rates level off asymptotically (Ali et al., 2003; Kozlowski, 1996). Indeterminate growth reduces the pressure to achieve optimal body size within a given time frame, especially if the fish are long‐lived and if territory establishment and breeding are not seasonally restricted. Rather than bearing the costs of faster‐than‐normal growth after incurring a temporal growth depression (“compensatory growth” sensu Hector & Nakagawa, 2012, where growth exceeds the normal, body size‐specific rate), fish that are not subject to seasonality‐ or life span‐induced time stress may continue growth at a normal size‐specific rate once conditions improve (“catch‐up growth,” Hector & Nakagawa, 2012). However, when juvenile fish are exposed to size‐selective predation, for example, by gape size‐limited piscine predators (Sogard, 1997), rapid compensatory growth may be favored despite its potential adverse side effects in order to outgrow the predation risk (Dmitriew, 2011; Metcalfe & Monaghan, 2003; Urban, 2008).

**FIGURE 1:**
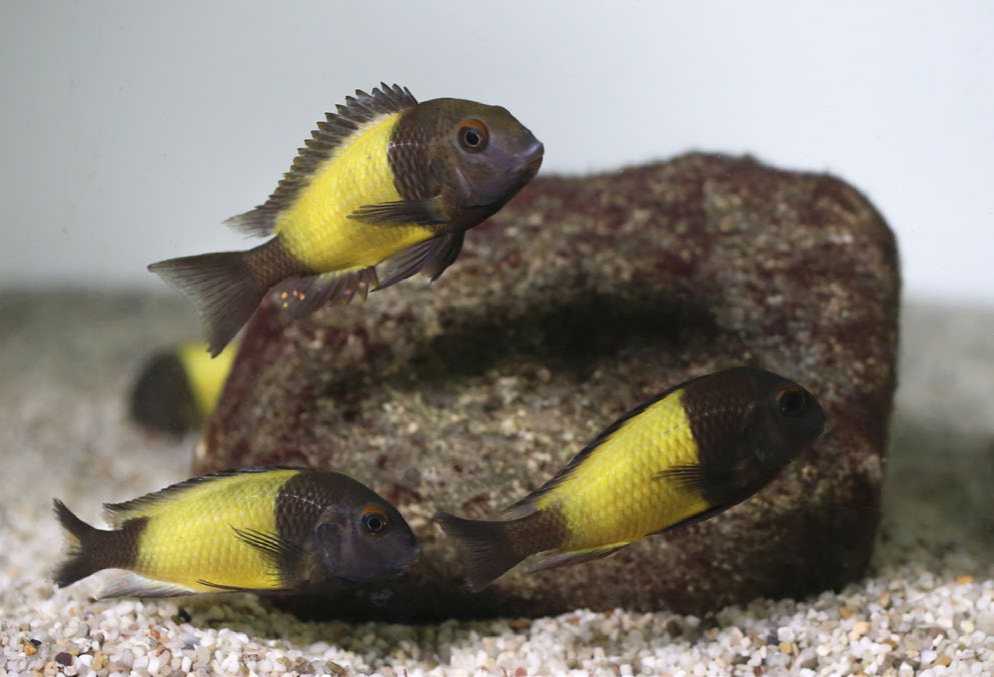
*Tropheus* sp. black “Ikola.” With fins erect, the top left fish prepares to defend “his” rock against two visiting tank mates. Photograph by Wolfgang Gessl, Institute of Biology, University of Graz (www.pisces.at)

In the present study, we investigate the effects of early‐life food deprivation on growth trajectories, body condition, and adult contest performance in a long‐lived tropical fish species. Both males and females in the genus *Tropheus* (Cichlidae) engage in competitive contests to establish and maintain their individual territories in the shallow rocky littoral of Lake Tanganyika, Africa. Territories supply food (epilithic algae), shelter, and—in case of males—mating opportunities (Hermann et al., 2015; Yanagisawa & Nishida, 1991). Males and females are similar in body size and display sexually monomorphic, conspicuous color patterns. In both sexes, success in contest competition for territories is positively correlated with body size (Odreitz & Sefc, 2015), and female preferences for males with high‐quality territories—that is, territories furnished with large rock structures—may indirectly select for large body size in males (Hermann et al., 2015). There is no seasonality in territoriality and breeding. Mating involves a temporary pair formation, as females move into their mates' territories for several days to weeks before spawning (Yanagisawa & Nishida, 1991). Female brood size increases with body size and weight (Schürch & Taborsky, 2005; KMS, pers. obs.). After spawning, females leave their mates to provide uniparental care by mouthbrooding their eggs and fry for about 30 days (Yanagisawa & Sato, 1990). During that period, females do not ingest any food, but pick up algae to supply the young in their mouths (Schürch & Taborsky, 2005; Yanagisawa & Nishida, 1991). Free swimming young (1.5 cm standard length, SL) are released in the very shallow littoral, where they hide at the bottom of boulders or between the small pebbles. Up to a body size of 4 cm (SL), the young *Tropheus* are typically found in depths of 2–3 m (Taborsky, 1999; Yanagisawa & Nishida, 1991). The density of aufwuchs feeders in the shallow rocky littoral is high (Takeuchi et al., 2010), and competition for food may expose the young *Tropheus* to nutritional deficits. Additionally, numerous fish of various trophic guilds prey upon young juveniles, and predator evasion behavior of young *Tropheus* may interfere with their foraging activity (Milinski, 1993; Urban, 2007). Free‐living *Tropheus moorii* grow rapidly during their first three years of life before their growth rate levels off at body sizes of 8–13 cm SL (Egger et al., 2004). In captivity, *Tropheus* start to reproduce at an age of 1–2 years (authors' pers. obs.), but age at first breeding may be higher in the wild. Otolith‐based aging of free‐living adults estimated an average age of 4 years in a population in southern Lake Tanganyika, with a maximum age of 10 years (Egger et al., 2004). Their longevity and the absence of a defined breeding season in the tropical lake suggests that with regard to lifetime reproductive success, temporarily growth‐restricted *Tropheus* should rather invest extra time in reaching competitive body size than risk the potential costs of accelerated growth (Hector & Nakagawa, 2012). However, juvenile *Tropheus* are exposed to size‐selective predation by an abundance of spiny eels (Mastacembalidae), small claroteid catfish, and other gape‐limited predatory fish in the shallow littoral (Abe, 1997; Koblmüller, 2021, pers. communication), which may promote a compensatory growth response in order for the juveniles to reach a body size refuge from these predators more quickly. To determine the growth strategies and long‐term consequences following early‐life food stress, we exposed young of *Tropheus* sp. “black” population “Ikola” (from here on referred to as *Tropheus* “Ikola”; Figure 1) to a period of intermittent feeding, which reduced their growth rate compared to a control group. We predicted that the food‐restricted fish would catch up in body size with the control group in order to avoid the disadvantages in adult competitive success and fecundity that are associated with small body size (Barneche et al., 2018; Koops & Grant, 1993; Odreitz & Sefc, 2015; Pincheira‐Donoso & Hunt, 2017). By relating growth rates to body size, we tested whether they did so by catch‐up growth, thereby avoiding potential costs of faster‐than‐normal growth, or compensatory growth, thereby quickly outgrowing size‐selective predation. After fish had matured into their adult color pattern, we staged experimental contests for territories to test whether performance in adult contest competition was affected by food restrictions during early life.

## 2 MATERIAL AND METHODS

### 2.1 Breeding and rearing conditions

The parental population consisted of wild‐caught individuals of *Tropheus* “Ikola” that had been purchased from an ornamental fish trader. For breeding, the fish were housed in pairs (if not aggressive toward their partner) or in groups of one male and several females (to avoid aggression) between April 2017 and July 2018. Some individuals bred more than once, but never with the same partner. Mothers were allowed to mouthbrood their broods for 2 weeks, while they were separated from their tank mates by mesh partitions. Then, each brood was transferred to an aerated fish breeding box (17 × 12.5 × 13.5 cm L × W × H). When all of the fry of a brood had absorbed their yolk sac (20–27 days of age postfertilization), they were transferred to a tank (60 × 30 × 30 cm L × W × H), using one tank per full‐sib family. At all times, the water temperature was kept between 25 and 26℃, and tanks were filtered with internal box filters and illuminated with an overhead white light on a 13‐hr:11‐hr light/dark cycle. We raised 14 broods with brood sizes ranging from 2 to 15 juveniles (99 juveniles in total). Females were identified based on their swollen genital papillae, which started to become apparent at about 4 cm SL (min. 32 weeks of age). Sexing was attempted or confirmed, respectively, at each time point when the fish were measured for size and weight (see below). By the end of the study period, 84 individuals had been reliably sexed and were included in the growth analyses. Housing and handling of the fish was covered by permit number BMWFW‐66.007/0004‐WF/V/3b/2016 issued by the Federal Ministry of Science, Research and Economy of Austria. The experiments were carried out with the ethical approval of the ethics committee of the University of Graz (permit number GZ. 39/45/63 ex 2019/20).

### 2.2 Feeding treatment

After 2 months in the full‐sib tanks, we applied a split‐brood design and assigned one half of each full‐sib family to the intermittent feeding (IF) group and the other half to the control group. At this point, the fish were 11–12 weeks old. The fish were then housed individually. To this aim, tanks of 60 × 30 × 30 cm (L × W × H) were separated by aluminum micromesh (grid size 1 × 1 mm) into 8 compartments of 15 × 15 × 30 cm. An aquarium filter was placed in one of the compartments, and the other seven compartments were stocked with one juvenile each. Chemical, acoustic, and visual interaction was possible across compartments. The water was changed weekly. All juveniles in a tank belonged to either the control or the IF group.

Individuals of the control group (*N* = 43) were fed ad libitum once a day in the morning, 5 days a week from Monday to Friday. Individuals in the IF group (*N* = 41) were also fed ad libitum, but only 3 days a week (Monday, Wednesday, and Friday), for a period of 20 weeks. By the end of the treatment period, the fish were approximately 8 months old, showed territorial behavior toward their compartment neighbors, and started to develop their adult color pattern. From then on, all fish received the same feeding regime (5 days a week, ad libitum) until the end of the study period. When a fish had reached a body size of 6.2–6.5 cm SL, it was transferred into a larger compartment (15 × 30 × 30 cm L × W × H).

### 2.3 Size and weight measurements

We documented body size and weight of all juveniles at the beginning of the treatment period (week 0) and every 4 weeks thereafter over a period of up to 80 weeks (21 measurement points). Prior to feeding that day's ration, juveniles were captured with small aquarium nets and gently laid on a wetted plastic board furnished with a ruler. Standard length (SL) was measured to the nearest 1 mm. Then, the fish were weighed (to the nearest 10 mg) in a cup of water placed on a scale.

Growth rates (GR, per 4‐week interval) were calculated as 100 × (ln SL_2_ − ln SL_1_), where ln SL_1_ and ln SL_2_ is the (natural) log‐transformed standard size at the beginning and the end, respectively, of each interval. Fulton's condition factor (Ricker, 1975; see also Nash et al., 2006) was calculated as *K* = 100 × W/SL^3^, with weight W in g and SL in cm.

The decreasing sample sizes toward the later measurement points (Table 1; from week 56 onward) do not reflect mortality, but are mainly due to the fact that we did not continue to monitor size and weight trajectories of the latest born fish for the full 80 weeks. This was because the IF fish had caught up with the controls and were prepared for the contest experiment.

**TABLE 1:** Average body size (mean SL, in cm) and Fulton's condition factor (mean *K*) of males and females in the control (C) and intermittent feeding (IF) groups measured at 4‐week intervals

Week	Group	Females						Males					
		*N*_SL_	Mean SL	*p* (ΔSL)	*N_K_*	Mean *K*	*p* (Δ*K*)	*N*_SL_	Mean SL	*p* (ΔSL)	*N_K_*	Mean *K*	*p* (Δ*K*)
0	C	13	2.28	.391	10	3.53	.845	30	2.24	.999	24	3.48	.418
	IF	17	2.35		11	3.52		24	2.23		21	3.40	
4	C	13	2.55	.069	13	3.33	.675	28	2.56	2.32 * 10^–5^	28	3.34	.934
	IF	17	2.48		17	3.28		24	2.34		23	3.33	
8	C	13	2.73	.**003**	13	3.48	.**014** ^a^	30	2.73	7.72 * 10^–7^	30	3.60	.081
	IF	17	2.52		17	3.29		24	2.45		24	3.50	
12	C	13	2.98	.**001**	13	3.44	.942	30	3.05	1.33 * 10^–10^	30	3.55	.584
	IF	17	2.64		17	3.41		24	2.64		24	3.51	
16	C	13	3.17	6.25 * 10^–5^	13	3.54	.680	30	3.31	2.52 * 10^–12^	30	3.63	.155
	IF	17	2.74		17	3.51		24	2.80		24	3.54	
20	C	13	3.47	1.43 * 10^–6^	13	3.53	.833	30	3.64	4.75 * 10^–15^	29	3.57	.469
	IF	17	2.94		17	3.48		24	3.05		23	3.55	
24	C	13	3.70	.**002**	13	3.57	.739	30	4.02	1.47 * 10^–11^	29	3.49	.202
	IF	17	3.25		17	3.61		24	3.42		24	3.61	
28	C	13	3.89	.**001**	13	3.49	.247	30	4.30	3.51 * 10^–11^	30	3.38	.080
	IF	17	3.47		17	3.58		24	3.75		24	3.49	
32	C	12	4.06	.**009**	12	3.42	.**007** ^a^	29	4.55	3.17 * 10^–10^	29	3.41	.146
	IF	17	3.72		17	3.51		24	4.05		24	3.52	
36	C	12	4.22	.**042** ^a^	12	3.54	.321	29	4.79	5.52 * 10^–9^	29	3.39	.105
	IF	17	3.96		17	3.57		24	4.32		23	3.47	
40	C	11	4.38	.**048** ^a^	11	3.43	.**017** ^a^	29	4.99	5.31 * 10^–7^	29	3.39	.168
	IF	17	4.16		16	3.62		24	4.55		23	3.45	
44	C	12	4.57	.192	12	3.51	.564	30	5.21	3.31 * 10^–5^	30	3.32	.064
	IF	17	4.41		17	3.53		24	4.90		24	3.36	
48	C	11	4.64	.243	11	3.51	.265	29	5.36	1.15 * 10^–4^	29	3.39	.757
	IF	17	4.52		17	3.59		24	5.05		24	3.34	
52	C	11	4.76	.567	11	3.44	.624	29	5.58	7.45 * 10^–6^	29	3.27	.102
	IF	14	4.66		17	3.47		23	5.22		23	3.34	
56	C	9	4.78	.749	9	3.49	.537	28	5.73	9.41 * 10^–5^	28	3.25	.865
	IF	12	4.74		12	3.48		22	5.40		22	3.24	
60	C	8	4.74	.342	8	3.57	.339	22	5.81	.**017**	22	3.33	.377
	IF	10	4.84		10	3.47		18	5.61		17	3.27	
64	C	9	4.88	.308	9	3.55	.752	23	5.91	.080	23	3.27	.666
	IF	12	5.02		12	3.45		19	5.77		19	3.27	
68	C	9	4.97	.588	9	3.50	.859	19	5.89	.410	19	3.39	.123
	IF	11	5.02		11	3.46		19	5.84		19	3.29	
72	C	8	4.95	.237	8	3.62	.428	26	6.09	.076	25	3.36	.147
	IF	10	5.08		10	3.53		23	5.96		23	3.26	
76	C	8	5.08	.550	8	3.50	.503	18	6.29	.**011**	18	3.28	.072
	IF	9	5.14		9	3.49		17	6.02		17	3.32	
80	C	4	5.10	.479	4	3.62	.630	13	6.13	.781	13	3.37	.835
	IF	7	5.19		7	3.57		7	6.09		7	3.35	

Note

*p* (ΔSL) and *p* (Δ*K*) are the *p*‐values for the difference in SL and *K*, respectively, between treatment groups, and were estimated by linear mixed models accounting for full‐sibship among individuals (see Methods; see Table S1 for a complete report of the LMM outputs). Significant *p*‐values (*p* < .05, without correction for multiple testing) are printed in bold (but see footnote a). *N*_SL_ and *N_K_*, number of fish in each group for comparisons of SL and *K*, respectively (lower sample size for *K* due to missing weight data).

^a^ Not significant after Benjamini–Hochberg correction for multiple testing. The correction was applied to each suite of data points separately (SL in females, *K* in females, SL in males, *K* in males).

### 2.4 Contest experiment

Contest trials were staged between body size‐ and sex‐matched IF and control feeding group fish that were selected to originate from different full‐sib families. Trials took place from August to November 2019. For the 30 IF fish, which were used in this experiment, this was approximately 13 to 23 months after the end of their intermittent feeding treatment (mean ± *SD* = 19 ± 3 months). The average body size of female contestants was 5.7 ± 0.2 cm SL (mean ± *SD*, range 5.4–6.2 cm) and that of male contestants was 6.6 ± 0.6 cm SL (range 5.4–7.7 cm). The average size difference between contestants was 0.08 ± 0.06 cm SL (mean ± *SD*) with a maximum of 0.2 cm SL, for females, and 0.15 ± 0.21 cm SL with a maximum of 0.8 cm SL for males. The number of trials that were conducted in this experiment was restricted by the number of size‐matched pairs that could be formed for each sex. After discarding four trials in which no clear dominance relationship was established, the data comprised 10 female–female and 20 male–male contest trials. Each fish partook in only one trial.

In order to control social conditions experienced by fish before entering the contest trial, the fish were housed with only one neighbor in the same tank for two weeks. We again used aluminum micromesh (grid size 1 × 1 mm) to divide 60 × 30 × 30 cm tanks into two partitions, and stocked each tank with one large and one small fish of the same sex. Body size determines dominance hierarchies in *Tropheus* (Odreitz & Sefc, 2015), and observations of the fish confirmed that the larger of the two fish displayed dominant behavior (lateral displays with erect fins directed at the smaller fish, charges toward the mesh partition separating the two fish) toward their smaller neighbors. Contests were always staged between individuals that had experienced the same social (dominant or subdominant) status during this period. On the day before the contest, body size and weight of contestants were determined as described above.

The setup of the contest trials followed Ziegelbecker et al. (2018). In short, we set up a contest arena (46 × 50 × 42 cm L × W × D), which was divided in half by a mesh partition (grid size 0.5 × 0.5 cm) and an opaque plastic board. Concrete bricks were placed next to the partition on each side, which allowed each fish to acclimate and occupy the brick structure within its half of the arena without disturbance from its neighbor, during a period of 3 hr. Then, the plastic board was removed, allowing visual contact between the contestants. After one more minute, removal of the mesh partition turned the previously subdivided brick construction into one continuous structure. The experimenter (nonblinded) observed the tank from a position hidden from the fish and ended a trial when a clear dominance relationship had been established, or after an observation time of 20 min (these four trials were discarded as unresolved). Typically, dominance relationships were resolved quickly and were considered to be established when one fish (the “winner”) occupied the brick construction and exhibited dominant body coloration, whereas the subordinate one sought shelter behind the brick structure or behind a board that was provided for that purpose and exhibited subordinate coloration for at least 3 min (see supplementary information of Ziegelbecker et al., 2018). Enduring aggression or harmful fights occurred in none of the contests; thus, no trial was ended prematurely. After the contest experiment, the fish were housed in groups in large tanks.

### 2.5 Statistical analyses

All analyses were carried out in R, version 4.0.2 (R Core Team, 2020) using the packages “lme4” (Bates et al., 2015) and “lmerTest” (Kuznetsova et al., 2017) to fit linear models, “mgcv” (version 1.8‐34; Wood, 2004, 2011) to fit generalized additive models, and “ggplot2” (Wickham, 2016) for plotting. The differences in body size (SL) and condition factor (*K*) between the IF and the control group and between the sexes were tested using linear mixed models (LMM) with treatment group or sex as fixed factor. To account for shared ancestry and shared early rearing conditions, full‐sib family (“brood ID”) was included as random factor. We checked for normality of residuals using Shapiro–Wilk tests. Significant deviations were detected in some of the LMMs for *K* and were found to be due to individual outlier values of *K*. Removal of these data points (1–5 per affected dataset) restored the normality of residuals (Shapiro–Wilk tests for residuals of all LMM with *p* > .05), while the effect estimates of the LMMs changed only marginally. Trajectories of growth rates and condition factors against time and SL were visualized by smoothed loess regression lines with 95% confidence intervals.

To compare the trajectories of growth rates and condition factor between the IF and the control group, we fitted generalized additive mixed models (GAMM) for each sex separately. We included treatment group as fixed factor, a smooth term for time or body size (time point, time interval or SL, dependent on which trajectories were compared) and a factor‐smooth interaction term (time by group, or body size by group), which tested for a difference in trajectories between IF and control fish. We added fish ID and brood ID as random factors, and a correlation term to account for autocorrelation within individual fish. An analogous GAMM was fitted to test for differences in the slopes of *K* over time between the sexes, using data from both treatment groups for each sex. We used the default “mgcv” setting of *k* = 10 to fit GAMMs to the complete 80‐week dataset and verified that results obtained with *k* set to 20 were almost identical. For the analysis of *K* across the treatment period (week 0–20), we varied *k* from 3 to 6 and report the model with the best fit (highest *R* ^2^_adj_.; again, the results were almost identical regardless of the value of *k*). To compare trajectories between treatment groups and sexes, we report the GAMM estimates for the factor‐smooth interaction terms and *R* ^2^_adj_. values for models including and excluding the interaction term (*R* ^2^_adj.,with_IA_ and *R* ^2^_adj.,without_IA_). In the absence of available routines for GAMM checking, we used the function *gam_check* to visualize the distribution of the residuals, but note that *gam_check* treats the random effects as part of the error and provides only approximate assessment of the fit of the mixed model. No evidence for model misspecification was detected in the *gam_check* output.

Finally, we used a generalized linear model (GLM) with a binomial error distribution to test whether the likelihood to win a contest differed between IF and control fish. The binary dependent variable coded the outcome of the dyadic contest from the perspective of the IF fish as “won” or “lost.” Although the contestants were size‐matched, we included relative size difference (RSD, calculated as (SL_IF fish_ − SL_control fish_)/(SL_IF fish_ + SL_control fish_)) as fixed factor to account for any remaining body size differences. We also included the time since the end of IF treatment as another fixed factor, as this varied among IF fish. Both continuous predictors were scaled and centered. Condition factor was not included in this analysis, as it had had no effect on contest outcome in previous experiments (Odreitz & Sefc, 2015; Ziegelbecker et al., 2018). Residual diagnostics were conducted using the R package “DHARMa” following the recommended workflow for binomial models (Hartig, 2021) and detected no evidence for model misspecification.

## 3 RESULTS

### 3.1 Body size trajectories

**FIGURE 2:**
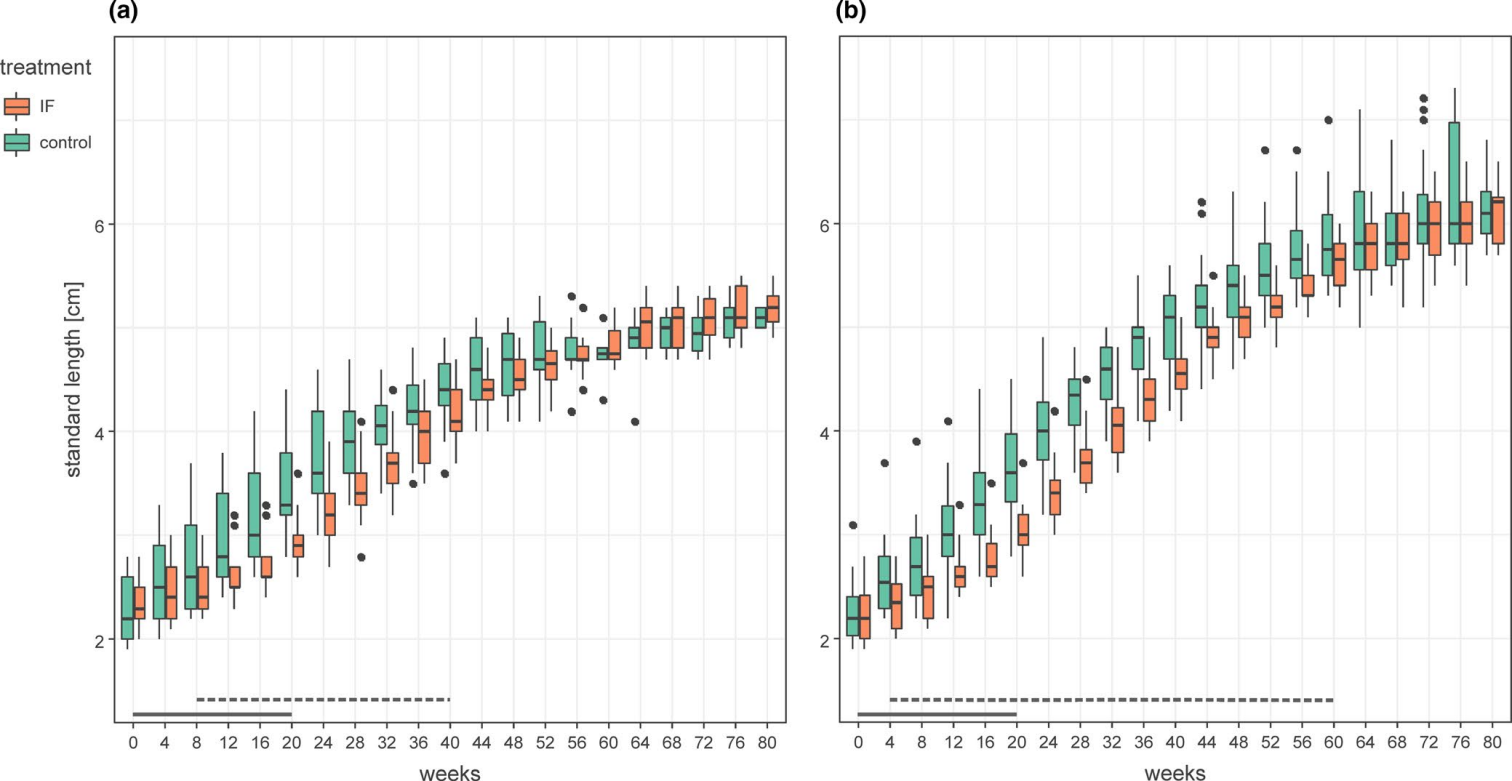
Body size of females (a) and males (b) of the intermittent feeding (IF) and the control group across the 80‐week study period. The duration of the IF treatment is indicated by solid lines (week 0–20). The horizontal broken lines mark the periods during which the body size of IF and control fish differed significantly from each other (see also Table 1)

**FIGURE 3:**
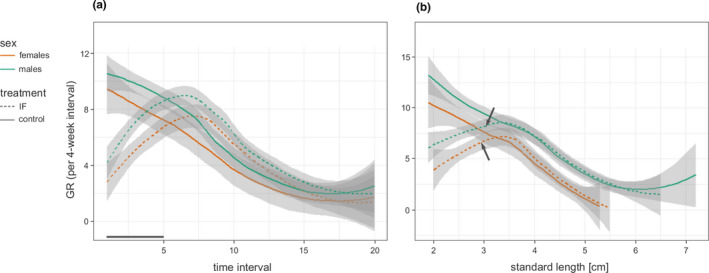
(a) Growth rates (GR) of males and females in the intermittent feeding (IF) and the control group plotted against time. The 4‐week time intervals are numbered, with interval 1 referring to growth between week 0 and week 4, etc. The solid line on the x‐axis indicates the duration of the IF treatment. In (b), GR is plotted against the standard length at the beginning of the corresponding 4‐week interval. The arrows mark the average SL of male and female IF fish at the end of the treatment period. The graphs show loess regression curves with 95% confidence bands. Sample sizes for each interval are given in Table S3

At the start of the treatment period, there were no statistically significant differences in body size between the IF and the control groups, nor between males and females across treatment groups (LMM, est_group_ = 0.024, *t* = 0.748, *p* = .457; est_sex_ = −0.017, *t* = −0.499, *p* = .62; sex‐specific treatment comparisons in Table 1, Table S1). Subsequently, growth rates were lower in females than in males (Figures 2 and 3a) and were analyzed separately for each sex. Fish in the IF group grew at lower rates than control fish (Figure 3a) and were significantly smaller than control fish after 4–8 weeks into the treatment (Figure 2; Table 1). At the end of the 20‐week treatment period, IF fish were 15% smaller than control fish (Table 1). The size differences between IF and control fish remained significant for a considerable amount of time (5–10 months) after the end of the treatment (Table 1; Figure 2). Males had fully caught up in size at an average body size of 5.85 ± 0.44 cm (mean ± *SD*) and females at 4.48 ± 0.29 cm.

### 3.2 Growth rate trajectories

The largest differences in growth rates between IF and control fish were observed in the first weeks of the treatment period (Figure 3a). In both sexes, growth rates of IF fish increased during and after the treatment period and reached their peaks two months after the end of the treatment, while growth rates of control fish decreased continuously (Figure 3a). The trajectories of growth rates over time revealed temporal differences between the treatment groups, as they differed significantly between IF and control fish in both sexes (Table 2A). After the end of the treatment period, growth rates of IF fish temporarily exceeded those of control fish, but did not surpass the high growth rates that were exhibited by the control fish at the beginning of the measurements (Figure 3a).

**TABLE 2:** GAMM estimates for factor‐smooth interaction terms for comparisons of the trajectories of growth rates and condition factors between IF and control fish

	edf	*F*	*p*‐value	*R* ^2^_adj.,with_IA_	*R* ^2^_adj.,without_IA_
(A) Growth rate against time					
Females	5.521	11.26	1.36 * 10^–10^	0.273	0.203
Males	3.899	16.89	5.31 * 10^–13^	0.308	0.265
(B) Growth rate against SL					
Females	4.006	12.54	9.06 * 10^–10^	0.283	0.247
Males	3.539	11.36	4.90 * 10^–8^	0.331	0.304
(C) Growth rate against SL (fish >3 cm)					
Females	1.001	0.151	.698	0.378	0.379
Males	1.000	0.18	.672	0.414	0.415
(D) Condition factor against time (during treatment period)					
Females	2.591	0.983	.224	0.039	0.045
Males	1.000	0.022	.882	0.060	0.063
(E) Condition factor against time (complete study period)					
Females	2.769	2.304	.153	0.026	0.018
Males	2.698	2.856	.097	0.134	0.126

Note

Significant *p*‐values and differences in model fit (*R* ^2^) indicate different trajectories.

Similarly, the trajectories of size‐specific growth rates—that is, growth rate plotted against body size—differed significantly between IF and control fish in both sexes due to the slow growth of IF fish at the beginning of the IF treatment (Table 2B; Figure 3b). Growth rates of IF fish approached those of the control fish at a body size of approx. 3 cm SL. From then on, IF fish continued to grow at similar rates as control fish of the same body size, and the decrease of growth rates with increasing SL was congruent in the IF and control group (GAMM with data of fish >3 cm SL, Table 2C; Figure 3b).

### 3.3 Body condition trajectories

**FIGURE 4:**
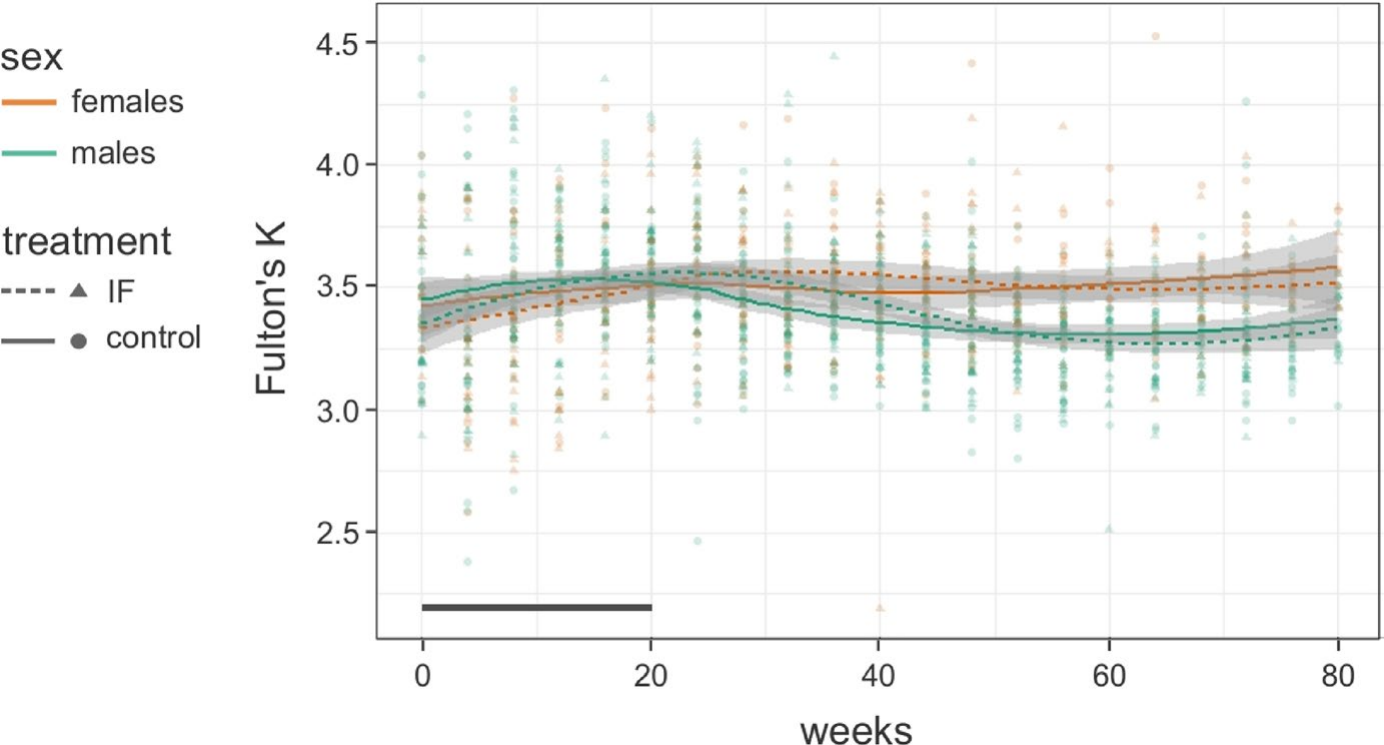
Condition factors (Fulton's *K*) of males and females in the intermittent feeding (IF) and control group across the 80‐week study period (scatterplot with loess regression curves and 95% confidence bands). The solid line on the x‐axis indicates the duration of the IF treatment (week 0–20)

Prior to the treatment, there was no difference in Fulton's *K* between males and females or between IF and control fish (LMM, est_sex_ = −0.103, *t* = −1.519, *p* = .135; est_group_ = −0.040, *t* = −0.626, *p* = .534; sex‐specific comparisons in Table 1, Table S1). Likewise, *K* values did not differ between sexes or between groups at the end of the treatment period (LMM, est_sex_ = 0.054, *t* = 1.186, *p* = .240; est_group_ = −0.022, *t* = −0.508, *p* = .613; sex‐specific comparisons in Table 1, Table S1). The trajectories of *K* over time did not differ significantly between treatment groups, neither during the IF period (Table 2D) nor across the complete experimental period (Table 2E). However, trajectories of *K* differed between the sexes (GAMM, interaction time and sex: edf = 4.78, *F* = 11.494, *p* = 3.4 * 10^–10^, *R* ^2^_adj.,with_IA_ = 0.110, *R* ^2^_adj.,without_IA_ = 0.073). Average *K* values of IF and control fish increased during the treatment period in both sexes, but, whereas *K* subsequently remained rather constant in females, it decreased in males (Figure 4).

### 3.4 Contest competition

The proportion of contests won by IF fish was very similar between males and females (11 out of 20 male–male, and 6 out of 10 female–female contests won by IF fish), and the sexes were pooled for further analysis. Neither time since IF treatment nor body size differences between contestants (RSD) had detectable effects on the IF fish's likelihood of winning the contest and IF fish were equally likely to win a contest as were control fish (Table 3).

**TABLE 3:** GLM analyzing the contest performance of IF‐treated fish against control fish

	Estimate	*z* value	*p*‐value
Intercept	0.293	0.771	.441
RSD	0.512	1.107	.268
Time since IF treatment	0.115	0.301	.763

Note

The nonsignificant intercept indicates equal chances of winning for both groups. Neither relative body size differences (RSD) between contestants nor time since the end of the IF treatment had a significant effect on contest outcome. RSD and time since IF treatment were scaled and centered.

## 4 DISCUSSION

With intermittent feeding of ad libitum rations over five months, our experiment simulated a mild but protracted food shortage. In the field, such conditions could be precipitated by a period of poor algal supply or strong foraging competition in the juveniles' habitat (McIntyre et al., 2006; Milinski, 1993) or elevated predation pressure interfering with foraging (Milinski, 1993). Female *Tropheus* invest heavily in the size and weight of their offspring, first by producing large eggs and then by providing buccal feeding to the mouthbrooded young (Schürch & Taborsky, 2005). This high parental effort was interpreted to be a response to limiting ecological conditions (Schürch & Taborsky, 2005) and suggests that in their natural environment, young *Tropheus* have to cope with variable food supply and perhaps other stressors such as adverse social environment and predation pressure. The intermittent feeding regime imposed in our study reduced the growth rate of the juveniles and resulted in body size deficits compared to the control group, which lasted for several months beyond the end of the IF treatment period. A meta‐analysis of growth responses to various types of dietary restrictions across a wide range of animals revealed that intermittent feeding was, on average, only moderately effective in inducing deficits in body length (Hector & Nakagawa, 2012). In this light, the effect of the IF treatment on body size observed in our experiment may be atypically strong, perhaps because the algivorous *Tropheus* are attuned to a more constant food supply, making an intermittent food supply more stressful than it would be for other trophic guilds. Both male and female IF fish caught up completely with the control groups, although males took longer to do so. Full compensation of body size deficits after experimental growth impairment has been reported for several fish species (food‐restricted salmon: Nicieza & Metcalfe, 1997; swordtails: Royle et al., 2005; stickleback: Ab Ghani & Merilä, 2015; Lee et al., 2016; gambusia: Livingston et al., 2014; killifish: Vrtílek & Reichard, 2015), while others, including females of a related cichlid species, failed to catch up completely after experiencing early‐life growth delays (cichlid, Taborsky, 2006; guppies, Auer et al., 2010; low temperature‐treated salmon, Nicieza & Metcalfe, 1997).

As pointed out previously (Hector & Nakagawa, 2012; Nicieza & Álvarez, 2009), the term “compensatory growth” has often been used to refer to a growth pattern, in which the food‐restricted group eventually reached the same final size as the control group, but either without comparing growth rates or without controlling for their size‐dependency (but see Ab Ghani & Merilä, 2015; Auer et al., 2010; Livingston et al., 2014). In the strict sense, compensatory growth refers to an acceleration of growth beyond the normal, size‐specific rate (Nicieza & Álvarez, 2009). In our experiment, IF fish grew at a higher rate than control group fish of the same age after being returned to standard feeding, but never at higher rate than control fish of the same size (Figure 3). Hence, there was no evidence for a compensatory growth response sensu Hector and Nakagawa (2012). Rather, IF fish made up for their early‐life growth deficits by growing at a normal, size‐specific rate (where “normal” refers to growth of the control group). This is in agreement with the hypothesis that the benefits of compensatory growth do not outweigh its potential costs in a species that exhibits indeterminate growth, has a long reproductive life span, and is not constrained by seasonality. In these circumstances, reduced longevity, which is a common corollary of accelerated growth, could have a stronger impact on total reproductive success than a temporary growth delay during juvenile development (Metcalfe & Monaghan, 2003).

The transition to the IF regime caused a significant drop in the growth rate that became apparent in the first measurement interval. Subsequently, the growth rates of the IF fish increased despite the food restriction and reached the normal, size‐specific rate at the end of the IF treatment period. The increase of the growth rate during the IF treatment suggests that food‐restricted juveniles invest in structural growth, which is consistent with the need to escape size‐selective predation by gape size‐limited piscine predators. Since the mass‐to‐length relationship of IF fish did not differ from that of control fish, their growth was apparently not achieved at the expense of body condition (discussed below). Rather, intermittent feeding may have allowed the IF fish to support simultaneous growth of structural and mobilizable tissue by hyperphagia on feeding days (Ali et al., 2003). As growth rates recovered during the treatment period, IF fish may also have acclimatized to the prolonged food restriction by adjusting their metabolic rate (Ali et al., 2003; O'Connor et al., 2000) or their digestive efficiency (Kotrschal et al., 2014).

Food deprivation during early life can have long‐lasting negative, species‐ and context‐specific effects on various fitness‐related traits, but it is often unclear whether these are due to the food deprivation itself or represent costs of subsequent compensatory growth (Dmitriew, 2011; Hector & Nakagawa, 2012; Metcalfe & Monaghan, 2001). In fish, faster‐than‐normal‐size‐specific growth following a period of food restriction was associated with an increase in the time to maturation in nine‐spined sticklebacks (Ab Ghani & Merilä, 2015) as well as with adult deficits in sexual attractiveness of male mosquitofish (Livingston et al., 2014) and litter size of female guppies (Auer et al., 2010). Following catch‐up growth at normal size‐specific rates after early‐life food restriction, male three‐spined sticklebacks and swordtails showed deficits in locomotor performance and reduced competitive ability as adults, as well as accelerated senescence (Álvarez & Metcalfe, 2007; Lee et al., 2016; Royle et al., 2005). Temporarily food‐restricted juvenile salmon first caught up with controls in weight and size, but later exhibited reduced growth and size‐specific mass (a proxy for lipid reserves) and deferred sexual maturation (Morgan & Metcalfe, 2001). In contrast, for the length of our experiment, growth rates of IF fish were not reduced below normal size‐specific levels once standard feeding was resumed, and size‐specific mass was not affected by feeding regimes. Furthermore, success in contest competition later in life was independent of whether or not fish had been exposed to the IF treatment as juveniles. Hence, compared to the controls, the IF fish suffered no deterioration of any of the potentially fitness‐related traits that were monitored in this experiment after the food restriction had been lifted. We note that the evidence for correlations between the measured traits and fitness is mostly indirect and deduced from observations that adult body size in *Tropheus* is correlated with the number of offspring spawned by a female (Schürch & Tab orsky, 2005), as well as with success in experimental territorial competition (Odreitz & Sefc, 2015) and the quality (structural composition) of the territory in the field (Yanagisawa & Nishida, 1991). Structural composition of the territory, in turn, is linked to food supply and shelter under field conditions (Yanagisawa & Nishida, 1991) and to male mating success under both field and experimental conditions (Hermann et al., 2015; Yanagisawa & Nishida, 1991). Finally, size and weight of juveniles were shown to be correlated with burst swimming speed that is relevant in predator escape (Schürch & Taborsky, 2005).

Body size‐specific mass, measured, for example, by Fulton's condition factor (*K*), is a proxy for energy reserves and hence considered a proxy for fitness of fish (Dmitriew, 2011; Mozsár et al., 2015), although the correlation between the density of lipids, which constitute the most important energy resource of fish, and *K* varies among species and seasons (Mozsár et al., 2015). A positive relationship between *K* and lipid density was for instance observed in bluegill sunfish, where *K* was also correlated with parasite load and reproductive success (Neff & Cargnelli, 2004). In other fish species, condition factors of individuals were related to swimming performance (Lapointe et al., 2006; Martínez et al., 2003; Romão et al., 2012), thermal tolerance (Robinson et al., 2008), early survival during settlement on reefs (Booth & Hixon, 1999) and stress response (Cook et al., 2012). During growth, individuals invest their resources into gains of structural size and mass, that is, nonmobilizable as well as mobilizable energy (Dmitriew, 2011), and may face an allocation problem when size and mass affect different fitness components. For instance, juvenile salmon continued skeletal growth during a phase of food restriction at the expense of size‐specific mass (Morgan & Metcalfe, 2001; Nicieza & Metcalfe, 1997). After the end of the food restriction period, the lipid reserves were restored quickly while the body size deficits were only partly compensated, consistent with the importance of lipid reserves for overwintering (Morgan & Metcalfe, 2001). Declines in condition factors during experimental food restriction of growing fish were also reported in other species (Auer et al., 2010; Booth & Hixon, 1999). In contrast, when exposed to intermittent feeding in our experiment, juvenile *Tropheus* maintained their condition factor at the same level as the control fish, but reduced their structural growth considerably. This is consistent with the hypothesis that animals have an “ideal” reserve‐to‐structure ratio which they seek to preserve (Broekhuizen et al., 1994). The observed trajectories of mass and size in food‐deprived juvenile *Tropheus* might suggest that their survival in the field is more strongly dependent on body condition than on body size during early life, perhaps due to a relationship between body condition and burst swimming speed (Schürch & Taborsky, 2005). However, the condition factors of males started to decline when they reached their maximum size‐specific growth rate, which, for the IF males, occurred a few weeks after the end of the treatment period. Females, in contrast, maintained their condition factor at a rather constant level throughout the post‐treatment observation period. Females also exhibited lower growth rates than males both during and after the treatment period. Sex differences in the investment in structural size versus condition factor during the late juvenile period may reflect sex‐specific contributions of condition and body size to adult fitness. In both sexes, success in competition for territories depends on body size (Odreitz & Sefc, 2015) but not on body condition (re‐analysis of data from Odreitz & Sefc, 2015; Ziegelbecker et al., 2018). Male *Tropheus* require a territory to attract mating partners (Hermann et al., 2015) and may therefore be under pressure to reach a competitive size rather quickly, although the growth rates of food‐deprived juveniles suggest that they avoid compromising their lifetime reproductive potential by exceedingly rapid growth. In contrast to males, females can mate while they are smaller (although offspring number increases with weight; Schürch & Taborsky, 2005), but require high energy reserves for breeding, as they do not ingest food and suffer significant losses of body condition during mouthbrooding (Schürch & Taborsky, 2005; Yanagisawa & Sato, 1990).

In conclusion, juvenile *Tropheus* responded to experimental food restriction by reducing their growth rate while maintaining their mass‐to‐size ratio at the level observed in control fish. After lifting the restriction, they caught up completely with the body size of the control fish, which is relevant since adult body size regulates access to resources and female fecundity (Odreitz & Sefc, 2015; Schürch & Taborsky, 2005; Yanagisawa & Nishida, 1991). There was no evidence for a compensatory growth response, as food‐restricted fish never exceeded the normal, size‐specific growth rates, which caused them to lag behind the body size of the control fish for an extended period of time. This is consistent with the idea that the protracted juvenile period and aseasonality of territory establishment and breeding allow the fish to catch up after a temporary growth impairment without risking any costs of accelerated growth. Growth costs often culminate in reduced longevity, which could affect the lifetime reproductive output of the long‐lived *Tropheus* more than a prolonged growth delay during juvenile development. The long life span of *Tropheus* did not allow us to collect data on the longevity of IF and control fish, but we detected no evidence for other consequences of early‐life food restrictions that have been reported in fish, such as permanently reduced growth rates or condition factors and reduced competitive ability. Altogether, our data suggest that *Tropheus* are capable of coping with mild food stress during juvenile development. However, we had also hypothesized that juvenile growth trajectories might be evolutionarily attuned to escape gape size‐limited predation as quickly as possible. Although growth rate trajectories during the IF treatment suggested that food‐restricted juveniles invest resources in body size gains, they neither did so by compensatory growth nor at the expense of their body condition. This suggests that young juveniles do not exploit their full physiological growth potential to escape gape size‐limited predators, but may depend on body condition to implement alternative predation avoidance strategies.

## CONFLICT OF INTEREST

None declared.

## AUTHOR CONTRIBUTIONS

**Angelika Ziegelbecker:** Conceptualization (equal); data curation (lead); formal analysis (equal); investigation (lead); methodology (equal); validation (equal); visualization (equal); writing‐original draft (lead); writing‐review & editing (supporting). **Kristina M. Sefc:** Conceptualization (equal); data curation (supporting); formal analysis (equal); funding acquisition (lead); investigation (supporting); methodology (equal); project administration (lead); supervision (lead); validation (equal); visualization (equal); writing‐original draft (supporting); writing‐review & editing (lead).

## Supporting information

Table S1‐S4

## Data Availability

Data used in this manuscript are provided as supplementary material.
